# Mental health during COVID-19 through Online Photovoice (OPV) in China

**DOI:** 10.3389/fpubh.2026.1773369

**Published:** 2026-06-03

**Authors:** Yongzhong Jiang, Xiang Cheng, Ahmet Tanhan, Vincent T. Francisco, Ihsan Cagatay Ulus, Melissa Hauber-Ozer, Ruixue Zou

**Affiliations:** 1 College of Management Science, Chengdu University of Technology, Chengdu, China; 2 Chengdu University of Technology, Chengdu, China; 3 Department of Guidance and Psychological Counseling, Faculty of Education, Adıyaman University, Adıyaman, Türkiye; 4 The University of North Carolina at Greensboro, Greensboro, NC, United States; 5 The University of Kansas, Lawrence, KS, United States; 6 Department of Guidance and Psychological Counseling, Faculty of Education, Bartın University, Bartın, Türkiye; 7 University of Missouri, Columbia, MO, United States

**Keywords:** China, COVID-19, mental health, Online Interpretative Phenomenological Analysis (OIPA), Online Photovoice (OPV)

## Abstract

This study investigates the primary facilitators of and barriers to mental health among Chinese participants during the COVID-19 pandemic. We collected the participants’ experiences using the Online Photovoice (OPV) method and analyzed the data through Online Interpretative Phenomenological Analysis (OIPA) to identify key facilitators and barriers. The results of the study revealed 12 major facilitator themes, including social support (21.58%, *n* = 41), socialization (15.26%, *n* = 29), and scenery (14.21%, *n* = 27). Conversely, eight major barrier themes emerged, including restricted daily life (34.71%, *n* = 59), the economy (32.94%, *n* = 56), and epidemic prevention policy (27.12%, *n* = 41). We discussed the findings and provided suggestions for researchers, mental health providers, key authorities, and educators. Our findings provide important implications for intervening in the mental health of individuals in response to the negative impact of the COVID-19 pandemic. As the researchers and the partners helped us with the study, we keep sharing the results with key authorities and at academic platforms.

## 1 Introduction

The COVID-19 pandemic that erupted at the end of 2019 has had a huge impact on the lives, mental health, and well-being of people around the world (1, 2, 85–87). To curb the global spread of this virus, numerous restrictions (e.g., wearing masks, maintaining spatial distances of at least 1.5 meters, etc.) were imposed on the daily lives of people in various countries (3, 88, 89), which have led to very serious biological, psychological, social, spiritual, and economic problems which were reported through Online Photovoice (OPV) method (4, 5, 90, 94). A report released by the World Health Organization (WHO) in March 2022 shows that in the first year of the pandemic, the global prevalence rates of anxiety disorders and depression increased significantly by 25%, with females being more susceptible than males (6), and the pandemic aggravating deterioration in mental health among young people with existing mental health problems (7). Population-based surveys in both high-income countries (8, 9) and low- and middle-income countries (10) have shown that mental health deteriorated during the COVID-19 epidemic, with frequent occurrence of depression, fear, anxiety, insomnia, somatization disorder, posttraumatic stress disorder (PTSD), and other psychopathological problems (11–13).

The abrupt changes in the public environment, economic hardship, prolonged closure orders, and uncertainty about the future brought about by the COVID-19 pandemic greatly affected people’s mental health resources and resilience (12). In this context, it is important to examine facilitators of resilience as well as barriers to mental health. In a previous study, Turkish researchers noted that the main facilitators of mental health among Turkish students during COVID-19 included support from family and friends, spending time in nature, and reading books. On the other hand, the main barriers were restrictions on freedom, psychosocial and spiritual problems, and not being able to gather with friends and relatives (89). In addition, Nigerian researchers reported that factors such as social support, hobbies, feelings of physical comfort, and religious beliefs contributed to the mental health of the Nigerian population during the COVID-19 pandemic, whereas COVID-19 limitations, inadequate socialization, and others were barriers to their mental health (85).

### 1.1 Study purpose

The main objectives of our study consisted of the following: (a) to understand the most important mental health facilitators and barriers for Chinese participants during the COVID-19 period through Online Photovoice (OPV) method; (b) to examine the systemic levels to which the facilitators and barriers belonged from the unique perspectives of the Chinese participants; (c) to test the applicability of the OPV methodology to the Chinese scenario; and (d) to communicate the results of the present study to stakeholders to further leverage the role of facilitators and address barriers.

### 1.2 Research novelty and contributions

While the literature on mental health during the pandemic has grown substantially, the vast majority of existing studies rely on rigid, quantitative psychometric tools, such as traditional Likert-scale online surveys. These positivism-based approaches often fail to capture the nuanced, highly localized realities of affected populations. We did not come across any OPV research in China on any topics including mental health during the pandemic.

To address this methodological gap, the present study offers a significant improvement through the first large-scale deployment of the Online Photovoice (OPV) method in less-developed regions of China. To our knowledge, this is the first OPV paper in China. Unlike traditional epidemiological surveys, OPV democratizes the research process by positioning participants as active co-investigators rather than mere subjects. By capturing real-time visual phenomenology coupled with structured narrative reflection (the SHOWED model), this approach fundamentally improves upon existing paradigms, revealing localized coping mechanisms—such as micro-adaptations to specific epidemic prevention policies and community volunteering—that top-down clinical tools frequently overlook.

## 2 Related work

### 2.1 COVID-19’s effect on China

China was the first country to detect an outbreak of COVID-19, and from late 2019 to August 2023, China reported a cumulative total of 99,300,923 confirmed cases of COVID-19 to the World Health Organization (WHO), including 121,628 deaths (14). As of March 22, 2023, a total of 3,515,872,818 vaccine doses had been administered (14). China is the second largest country in the world in terms of population, making the task of interrupting the spread of the virus more arduous than in other countries (15). In response to this global crisis, the Chinese government and people have made tremendous efforts, taking measures such as locking down schools, cities, streets, and residential areas, stopping work and production, and conducting large-scale nucleic acid testing throughout the city (16), which has brought about huge economic losses. And although the nearly three-year-long epidemic control policy has effectively safeguarded the lives of the Chinese people, it has inevitably brought about serious psychological problems (17). A study of social text analyses on China’s largest social media platforms showed that the COVID-19 pandemic had a significant negative impact on people’s mental health (6). Furthermore, anxiety was the central topic of psychological help counseling during the first (February 28, 2020 to April 28, 2020) and second (April 29, 2020 to April 23, 2021) phases of the pandemic (18). Anger, sadness, and obsessive-compulsive symptoms increased during the second phase, whereas anxiety, somatization, fear, and feelings of stress decreased (18). Compared to the general population, mental health risks and symptoms of mental disorders tended to be higher among frontline healthcare workers and police officers during COVID-19 restrictions (19–23).

### 2.2 Online Photovoice (OPV) and our research

Online Photovoice (OPV) is a participatory action research method that enables people to document and reflect on the concerns of their communities and facilitate critical dialogue on important issues by sharing and discussing photographs (24, 25, 87). Caroline Wang developed the method, initially named *photo novella*, in the early 1990s as part of a larger study documenting women’s health-related perspectives and needs in China’s rural Yunnan (26). A review of the literature reveals that traditional photovoice studies with samples of Chinese people have focused on mobile populations (27, 28), patient care (29), expectations of older adults’ care (30–32), teacher training (33, 34), gender identity negotiation (35), and academic stress (36). Most of these studies have been concentrated in economically developed regions along the coast of China, whereas few photovoice studies have been conducted in the less-developed regions of China. For instance, caregivers in Hong Kong were invited to use the photovoice method to express how positive thoughts could help them manage the stress and burden of caregiving when caring for a young psychiatric patient (37).

Although photovoice (PV) methods have been utilized quite extensively over the past decade (2, 87, 90), there are some key limitations to traditional photovoice methods (38, 90). Gabrielsson et al. (39) noted issues of methodological fit (40), how participant voice influences design and dissemination (41), links to justice and change (42), and the ethics of fairness (38), among other issues. To empower participants and communities (90) developed the Online Photovoice (OPV) method based on traditional photovoice. The OPV approach has been applied to different topics and contexts: mental health and spiritual well-being (85, 90), online distance education (91), sexuality and intimate relationships (86, 92), specific learning disabilities (93), women dealing with infertility (25), teacher candidates’ education (4, 43, 44), nurses transitioning to practice (24), college students’ perception of tasawwuf (45) to name a few. Despite the diversity of research topics, in our literature review, we found no studies that used OPV approaches to discuss the mental health of participants in China during COVID-19. Through critical review and discussion, we concluded that the use of the OPV methodology could be of practical value in helping participants in China’s less-developed areas to empower them to explore and share their living conditions and to engage in discussion and work for change on important community issues (e.g., mental health).

Beyond abrupt changes in the social environment, the sudden shift to remote settings fundamentally altered daily operational structures, particularly in education. While online learning provided a vital lifeline for academic continuity, it simultaneously generated acute stress. Multinational evaluations of e-learning during the COVID-19 pandemic indicate that technical barriers, social isolation, and reduced teacher presence severely degraded the student learning experience, acting as a substantial barrier to mental health (46). Within the context of the current study, academic pressure and ineffective virtual learning environments similarly emerged as major stressors among the student population.

Furthermore, pandemic-induced physical isolation and anxiety not only caused psychological trauma but also triggered global disruptions in somatic and biological rhythms, most notably altering sleep architecture (87). Sleep is one of the most important aspect of health (47) and its function on overall functioning gets even more critical during difficult times like traumatic processes (87). Extensive public health surveillance shows that severe sleep-related consequences, such as insomnia and sleep apnea, deeply affected both non-hospitalized COVID-19 survivors and the general population enduring prolonged lockdowns (48, 49). Post-infection insomnia prevalence reached alarming rates and was often exacerbated by concurrent depressive and anxiety symptoms (49). The deployment of advanced machine learning classifiers further demonstrated that demographic factors, employment status, and physiological indicators like oxygen saturation were highly predictive of chronic insomnia during the pandemic (50). These biological disruptions interact cyclically with psychological distress, physiologically corroborating the mental health barriers highlighted by participants facing a “restricted daily life.”

### 2.3 Theoretical framework

This study employed a framework based on ecological systems theory (EST), Online Photovoice (OPV), and community-based participatory research (CBPR) (51, 85). To fully understand the facilitators of and barriers to mental health among participants during the COVID-19 pandemic protecting people’s experiences as they are and considering OPV being an innovative and not well-known research method, we strived to make the participation easy, practical and enjoyable. we used an online questionnaire to collect the relevant data needed for the OPV study (including photographs and stories). Participant collaboration was essential throughout the collection process, so we used CBPR to help participants understand our study better, providing a Qualtrics survey that included video, audio, and text so that the participants can choose one or more way that is the most useful and meaningful to them to understand the study and engage in the study while maximizing the preservation of their voices and experiences (52). Additional details on the framework can be found in (87) study.

## 3 Materials and methods

We designed each aspect of this study employing the framework mentioned above and used the OPV approach to understand the most important facilitators and barriers affecting participants’ mental health during the COVID-19 pandemic.

### 3.1 Participants

We conducted data collection by distributing an online Qualtrics questionnaire during the COVID-19 pandemic which invited participants to answer questions about facilitators of and barriers to mental health and provide demographic information. We used video, audio and text at every single section of the survey to make the participation very clear, easy, and enjoyable. Considering the novelty and length of the study, as the OPV developers (87, 90) suggested when working under difficult conditions, we asked for consent two times: a temporary one at the beginning and the final one at the end of the study. The first consent question was to inform them that we ask for consent and their final answer to the final consent question at the end of the survey will be considered. In total, based on the final consent question, 212 participants provided informed consent and effectively finished the questionnaire for this study. Among these participants, 190 participants effectively completed the facilitator section, 170 participants effectively completed the barrier section, and 95 participants effectively completed the demographic information section. We have provided detailed demographic information in Table 1.

**Table 1:** Demographic information.

Variable (s)	Categories	(*N* = 95)
Age in years (mean ± standard deviation)	–	27.58 ± 8.39
Gender (number of participants and %)	Male	43 (45.26%)
	Female	41 (43.16%)
	Prefer not to disclose	11 (11.58%)
Number of cities the sample was from	–	23
From the country or immigrant (number of participants and %)	From the country (native)	85 (89.47%)
	Immigrant	0 (0.00%)
	Prefer not to disclose	10 (10.53%)
Marital status (number of participants and %)	Single	56 (58.95%)
	Married	30 (31.58%)
	Other, including cohabiting	4 (4.21%)
	Prefer not to disclose	5 (5.26%)
Spiritual/religious tradition (optional question; number of participants and %)	Christian	0 (0.00%)
	Muslim	0 (0.00%)
	Buddhism	3 (3.16%)
	Taoism	0 (0.00%)
	Other or none	79 (83.16%)
	Left empty	13 (13.68%)
Socio-economic status (number of participants and %)	Low	48 (50.53%)
	Middle	19 (20.0%)
	High	28 (29.47%)
Level of education (number of participants and %)	Middle school or below	3 (3.16%)
	High school	8 (8.42%)
	College	73 (76.84%)
	Master/doctorate	11 (11.58%)
Students (number of participants and %)	(Undergraduate or graduate)	62 (65.26%)
Year of enrollment (number of participants and %)	1st	12 (12.63%)
	2nd	7 (7.37%)
	3rd	22 (23.16%)
	4th	6 (6.32%)
	5th	10 (10.53%)
	Not a student	38 (40.0%)
How many different majors	–	33
Job and profession are the same (number of participants and %)	Yes	72 (75.79%)
	No	23 (24.21%)
	Left empty	0 (0.00%)
Work in a healthcare setting (number of participants and %)	Yes	7 (7.37%)
	No	85 (89.47%)
	Prefer not to disclose	3 (3.16%)
Diagnosed with COVID-19 (number of participants and %)	Yes	87 (91.58%)
	No	7 (7.37%)
	Prefer not to disclose	1 (1.05%)
Housing during the pandemic (able to choose more than one option; number of participants and %)	House/apartment with a garden	12 (12.63%)
	House/apartment without a garden	71 (74.74%)
	Do not have a place to live	7 (7.37%)
	Other	5 (5.26%)

117 incomplete responses were excluded from analysis.

### 3.2 Procedure

We reviewed the literature on mental health, OPV, and the use of this approach during the COVID-19 outbreak. Public mental health is a core issue in public health initiatives (53); thus, there is a need to use OPV to understand the mental health of different categories of people during the COVID-19 pandemic in a more effective way (90). In line with the suggestions of the literature, we followed the necessary steps to obtain informed consent from the participants and launched the study.

### 3.3 Data collection tools: online questionnaire

We designed an online Qualtrics questionnaire that included informed consent form, a detailed explanation of OPV methodology and the form to participate, a demographic information form, and COVID-19-specific questions. Aligning with the OPV philosophy, the developer of OPV and previous OPV researchers, at the beginning of the study we provided and kindly and strongly suggested to watch, listen or read about OPV content that has been prepared and provided by the developer of OPV. For the video explaining OPV https://youtu.be/ArqgA33EMDQ?si=5mp7Dh3K4DOW02Vu.

#### 3.3.1 Informed consent and demographic information form

The informed consent form explicitly outlined the study’s purpose, the voluntary nature of their participation, and their absolute right to withdraw from the study at any time without penalty. Importantly, given the visual nature of the OPV method, the consent form specifically requested participants’ permission to use their submitted photographs and narratives for academic analysis and future publications, assuring them that no identifiable information would be disclosed. Additionally, the survey included demographic and COVID-19-specific questions (e.g., gender, age, nationality, religious beliefs, level of education, economic status, COVID-19 diagnosis).

#### 3.3.2 Video, audio, and written explanations of OPV

In each section of the study, we provided participants with three options (video, audio, and written files) to explain the OPV participation process. All three options included the same details and helped participants learn about how to engage in the study. These different options were provided to mitigate possible disadvantages and increase the participation of individuals with reading difficulties or poor internet connections, for example, in line with the theoretical framework of the study. Developed by (87), the first part explained the OPV method and how to meaningfully and effectively participate.

### 3.4 Online Photovoice (OPV) procedures

As outlined by (87), the participants followed five steps for identifying the most important facilitators and barriers. The participants first completed the five steps for the facilitators and the for the barriers. The first step, named *facilitators (support, strength)/barriers (concern, issue)*, required participants to list at least one and at most 10 important facilitators of and barriers to mental health during the COVID-19 pandemic in a text box provided in the questionnaire. The second step involved taking one or more photo(s) representing the most important facilitator and barrier. The third step comprised uploading photos and stories. This section asked participants to choose and upload the most representative photo based on their perception and decision. Then they utilized the SHOWED questions to write a story for the selected photo. Participants were explained the acronym as follows:

S: What do you *S*ee in the picture representing a facilitator for you or your community population’ mental health during the COVID-19 process? What do you *S*ee in the picture representing a barrier for you or your community’s online/distance education during the COVID-19 process?
H: What is *H*appening in your photograph/picture? (Briefly describe).
O: How does it relate to (y)*O*ur life or your community?
W: *W*hat is it that creates or contributes to this most important facilitator? *W*hat is it that causes, creates or contributes to this most important barrier?
E: What do you *E*xperience (feelings, thoughts, behaviors) while taking the picture, writing your message, and submitting them?
D: What can we (as mental health professionals, educators, researchers, peers, etc.) *D*o about this?

The fourth step was named *summary, theme(s), or metaphor(s)*. This section required participants to provide at least one and at most three summary words, themes or metaphors to summarize their photo and story.

In the fifth step was named *attribution of facilitators and barriers to ecological system theory (EST)*. This section asked participants to specify the levels of systems (individual or intrapsychic, microsystem, exosystem, macrosystem, or all) which contributed to the development of this facilitator and barrier from their unique perspectives.

### 3.5 Data analysis: Online Interpretative Phenomenological Analysis (OIPA)

Developed Online Interpretative Phenomenological Analysis (OIPA) specifically to analyze OPV in order to preserve the experiences of people and convey their message as they are and as people would like their voices to be conveyed to others and especially to the ones holding critical positions and making laws (87, 90). The authors developed OIPA based on the shoulder of Interpretative Phenomenological Analysis (IPA). It is a method of explaining how participants make sense of their personal and social perceptions of the world, particular events, and experiences (54). In our study, considering the subsequent effects of COVID-19, we utilized OIPA to analyze and elaborate on photos provided by participants. The approach is carried out in five steps: (1) doing primary analyses on data (e.g., photos, captions, themes, research form) for missing information, (2) strict ethical compliance and confidentiality (e.g., systematically screening for and excluding/blurring any photos identifiable human faces, private property, or geolocation tags to protect participants’ privacy), (3) classifying the facilitator themes, (4) classifying the barrier themes, and (5) analyzing how the students attributed the themes to EST levels. Furthermore, all collected digital data were stored on a secure, password-protected server accessible only to the core research team.

### 3.6 Ethical considerations

Our study was completely anonymous, online, non-clinical, and observational. We did not conduct any medical or mental health interventions nor collect identifiable personal data. We got formal approval from Institutional Review Board (IRB) of Bartın University in Turkey and The University of North Carolina at Greensboro in USA for global OPV mental health online studies including the study for China. The reason behind this was, the developer of OPV, one of the main leading project persons and one of the coauthors of this current study being affiliated with these two universities in these two countries. We followed strict adherence to the ethical principles of the Declaration of Helsinki. Prior to participation, all respondents were provided with detailed information regarding the study’s purpose, the Online Photovoice (OPV) methodology, and intended data usage. Explicit electronic informed consent was obtained from all 212 participants. To ensure privacy and confidentiality, the dataset was fully anonymized; specifically, no personally identifiable information—such as human faces or private property—was permitted in the submitted photographs. Furthermore, participants were informed that their involvement was entirely voluntary and that they retained the right to withdraw from the study at any time without penalty.

## 4 Results and analysis

Based on CBPR (51, 55, 87) principles, we analyzed the data based on the unique perspectives of the Chinese participants and organized the results into the following four main sections: (1) descriptions of contextual factors related to mental health during the pandemic, (2) facilitator themes of mental health during COVID-19, (3) barrier themes of mental health during COVID-19, and (4) attribution of the facilitator and barrier themes to EST levels. Our findings are in general agreement with those of other OPV studies on mental health facilitators and barriers during COVID-19 (Table 2).

**Table 2:** Descriptive statistics for contextual factors related to mental health during the pandemic (*N* = 95).

Questions or items: during the pandemic, to what level do you do the following? (0 “not at all” to 100 “completely”)	(Mean ± SD)
Identify as a spiritual person	48.29 ± 34.25
Identify as a religious person	24.20 ± 26.89
Find religiosity/spirituality important in your life	41.32 ± 33.45
Have access to the internet	82.86 ± 20.22
Have a personal computer or tablet	88.57 ± 25.35
Have a smartphone	95.31 ± 16.16
Use the internet to access mental health services or related information	59.59 ± 32.61
Access offline mental health counseling services	29.84 ± 31.95
Access online mental health counseling services	32.11 ± 33.00
Find satisfaction with received or available offline mental health counseling services	41.41 ± 35.09
Find satisfaction with received or available online mental health counseling services	43.58 ± 35.90
Practice social distancing	66.54 ± 30.69
Favor COVID-19 vaccination	82.53 ± 30.15
Favor COVID-19 vaccination for family members	82.36 ± 29.30

### 4.1 Facilitators

**Figure 1:**
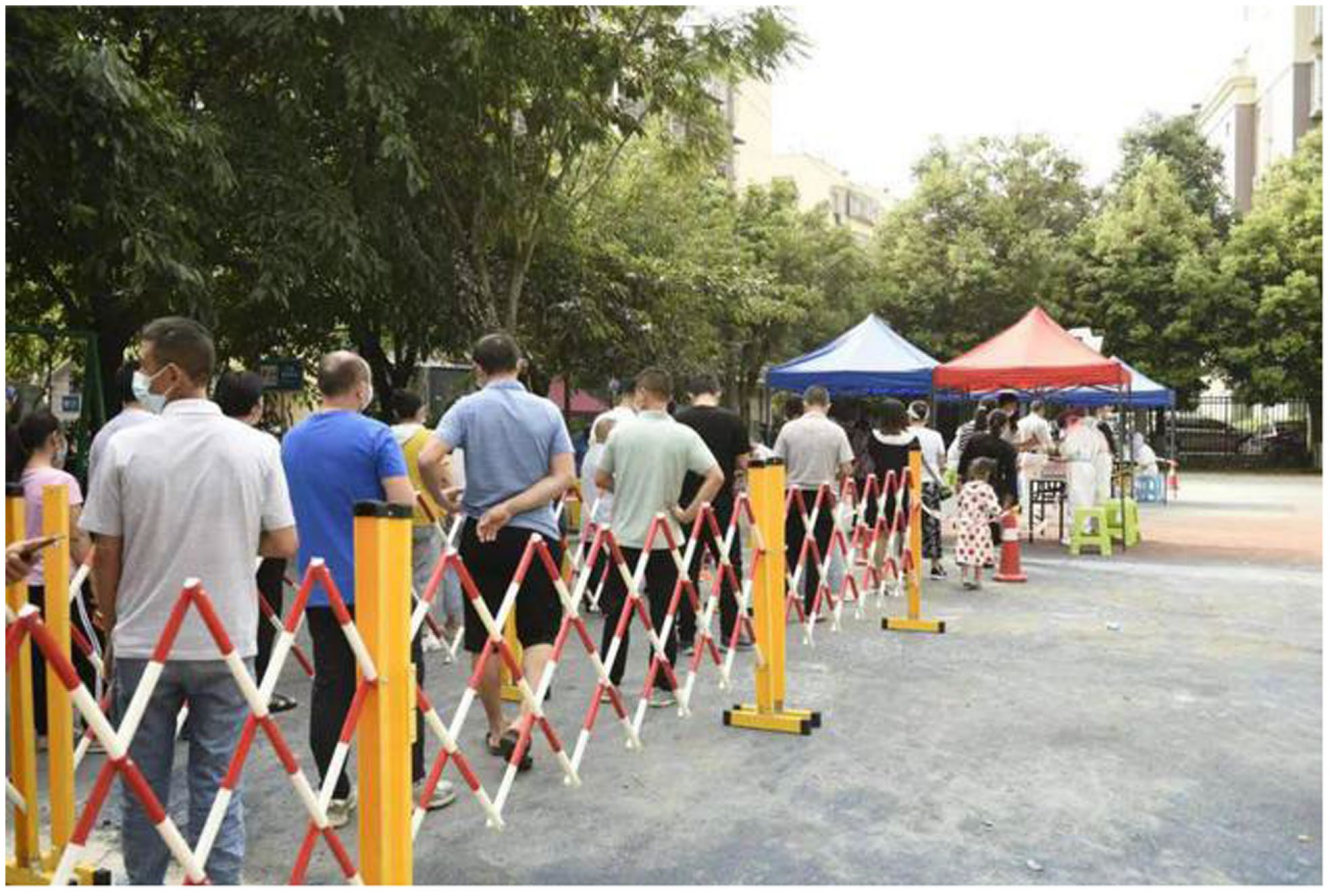
Dilemma, warmth, and kindness.

From the qualitative responses of 190 participants, we identified 12 key facilitator themes that positively influenced mental health during the COVID-19 pandemic. These themes, alongside their specific sub-categories and frequencies, are systematically summarized in Table 3. To provide deeper contextual understanding, participants submitted corresponding stories and photographs using the SHOWED model. These visual results act as phenomenological evidence, illustrating the lived experiences behind the themes. For instance, the photographs below capture specific instances of ‘social support’ and ‘government support,’ visually demonstrating how community resilience and frontline workers acted as primary mental health facilitators. Following the advice of the OPV developers, we preserved participants’ anonymity and only corrected minor spelling errors in their narratives to maintain authenticity (Figure 1).

**Table 3:** Facilitators of mental health during COVID-19 (*N* = 190).

Themes (12 main themes)	N	%
1. Social support	41	21.58
Nucleic acid detection		
Medical personnel		
Mobile health resources		
Harmonious and warm atmosphere		
Volunteer service		
Active cooperation of the masses		
Community support		
Selfless dedication of personnel from different fields		
-School support		
2. Socialization	29	15.26
Communication with friends		
The company of family members		
Working with coworkers		
Learning with classmates at school		
Sharing interesting things/ideas with others		
Interpersonal relationships		
Taking part in recreational activities		
Playing basketball with friends		
3. Scenery	27	14.21
Good weather has alleviated inner restlessness		
The charm of nature		
Difficulties become insignificant in the face of nature		
So happy to appreciate the beauty		
Memory		
4. Consciousness	26	13.68
Ideological awareness		
Self-reflection		
5. Gourmet food	24	12.63
Food brings inner satisfaction		
Supplemental nutrition		
Relieving stress		
Spiritual pleasure		
6. Entertainment	22	11.58
Diversion (bilibili, anime)		
Playing games		
Time-consuming		
Listening to music		
Reading books		
Movies		
7. Government support	22	11.58
Epidemic prevention and control policy		
The concept of putting the people first		
The determination and action of the government		
Beneficial to social development and stability		
8. Healthy lifestyle	22	11.58
Physical exercise		
Eating habits		
Daily routine		
9. Economic support	17	8.95
Quality of life		
Social status		
Industrial development		
10. Learning	14	7.37
Network resources		
Making plans		
Online learning		
Putting into practice		
Realizing one’s ideals and goals		
11. Animals and plants	9	4.74
Flowers make people feel happy		
Dogs/cats make people feel healed		
12. Media	8	4.21
Transmission of information (apps)		
Providing emotional value		

212 respondents; 22 invalid; 8 repetitions; 190 valid responses. We combined the themes represented in under 3% of the total number (190) of responses following the completion of analysis as suggested by (87) and (90).

A participant’s visual representation of community resilience, capturing residents queuing for nucleic acid testing. This image illustrates the facilitator theme of Social Support, specifically highlighting the positive psychological impact of observing order, mutual assistance, and role models within the community during pandemic lockdowns.

Participant 1 submitted the following story for the facilitator:

This image captures people queuing for nucleic acid during COVID-19. At that time, the hot topics in the news were mainly focused on “COVID-19,” “which city has an outbreak of the virus,” “which residential area has been closed again,” “those who go home across the region must be reported to the community, and those who come back from risky areas must be placed in quarantine,” “all residents must undergo nucleic acid testing,” and so on. However, amidst these challenges, I witnessed societal harmony, warmth, and genuine compassion. During the period of closure management, some generously shared living resources with neighbors, despite their hardships, which deeply moved me. In response, I’d like to say that with such role models around, I’d like to be one of those people who give kindness to others in tough circumstances.

Participant 1 submitted the following summary words for the photo and caption: Dilemma, Warmth, and Kindness.

**Figure 2:**
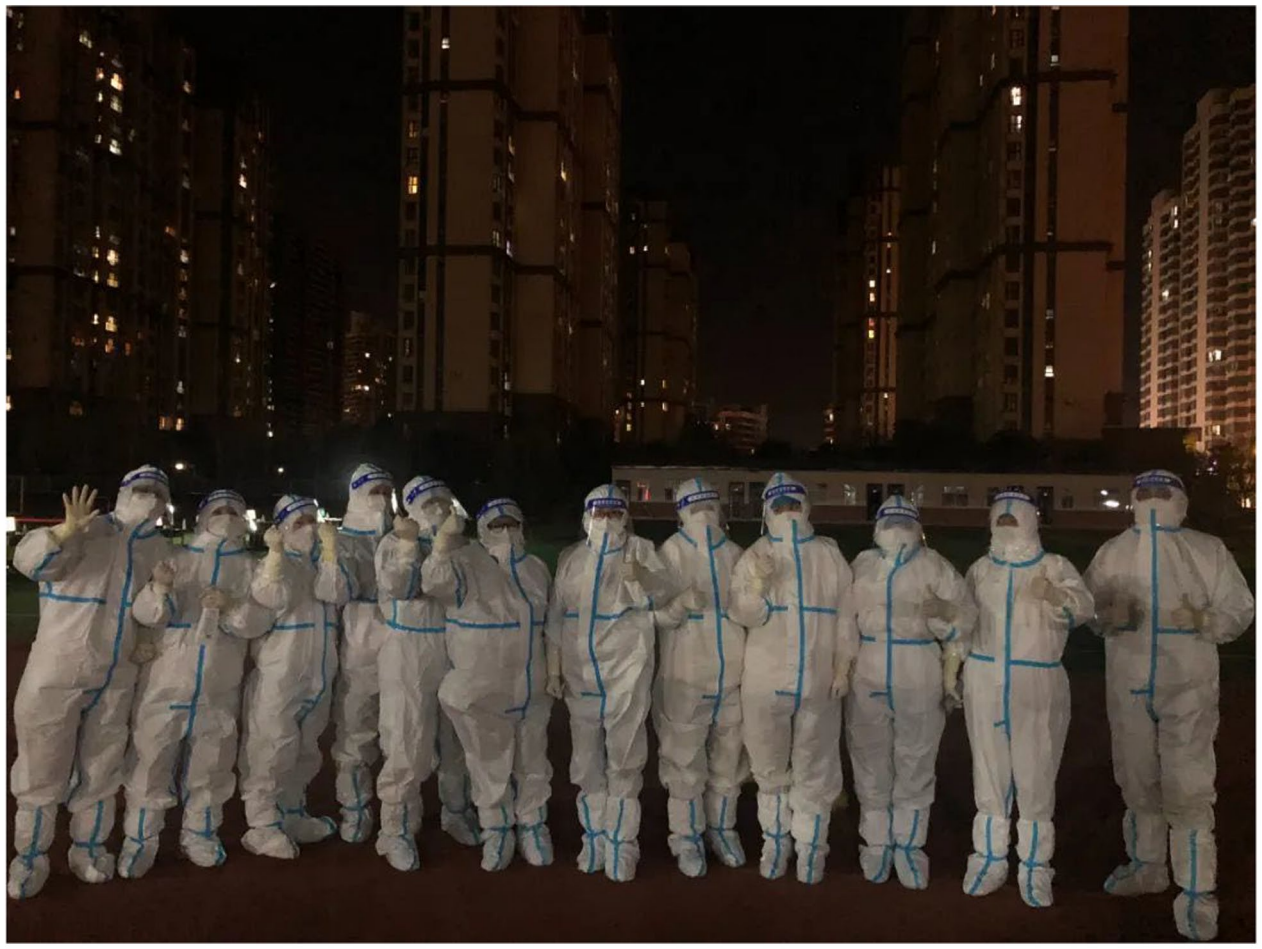
Epidemic prevention personnel, selfless dedication, and guarding lives.

This submitted photograph highlights the facilitator theme of Government Support and Social Support. The visual captures frontline medical workers in full protective gear at night, which the participant interpreted as a profound source of psychological safety, inspiration, and national safeguarding (Figure 2).

Participant 39 submitted the following story for the facilitator:

The picture is a group photo of medical personnel working to prevent the epidemic. In this war without smoke, a group of “soldiers” who “walk against the wind” made a letter of request to go to the front lines to support the emergency outbreak. They worked day and night, wearing a thick layer of protective clothing and large-sized diapers, with sweat running down from their clothes, blisters and broken skin on their hands, but they never complained of suffering or fatigue. During COVID-19, they always stood at the front line of the fight against the epidemic, displaying unwavering perseverance and courage. Even though I’m not acquainted with the epidemic prevention personnel in the picture, they are around each one of us! It is their selfless contribution that has brought us peace and safety!

Participant 39 submitted the following summary words for the photo and caption:

Epidemic prevention personnel, Selfless dedication, and Guarding lives.

### 4.2 Barriers

**Figure 3:**
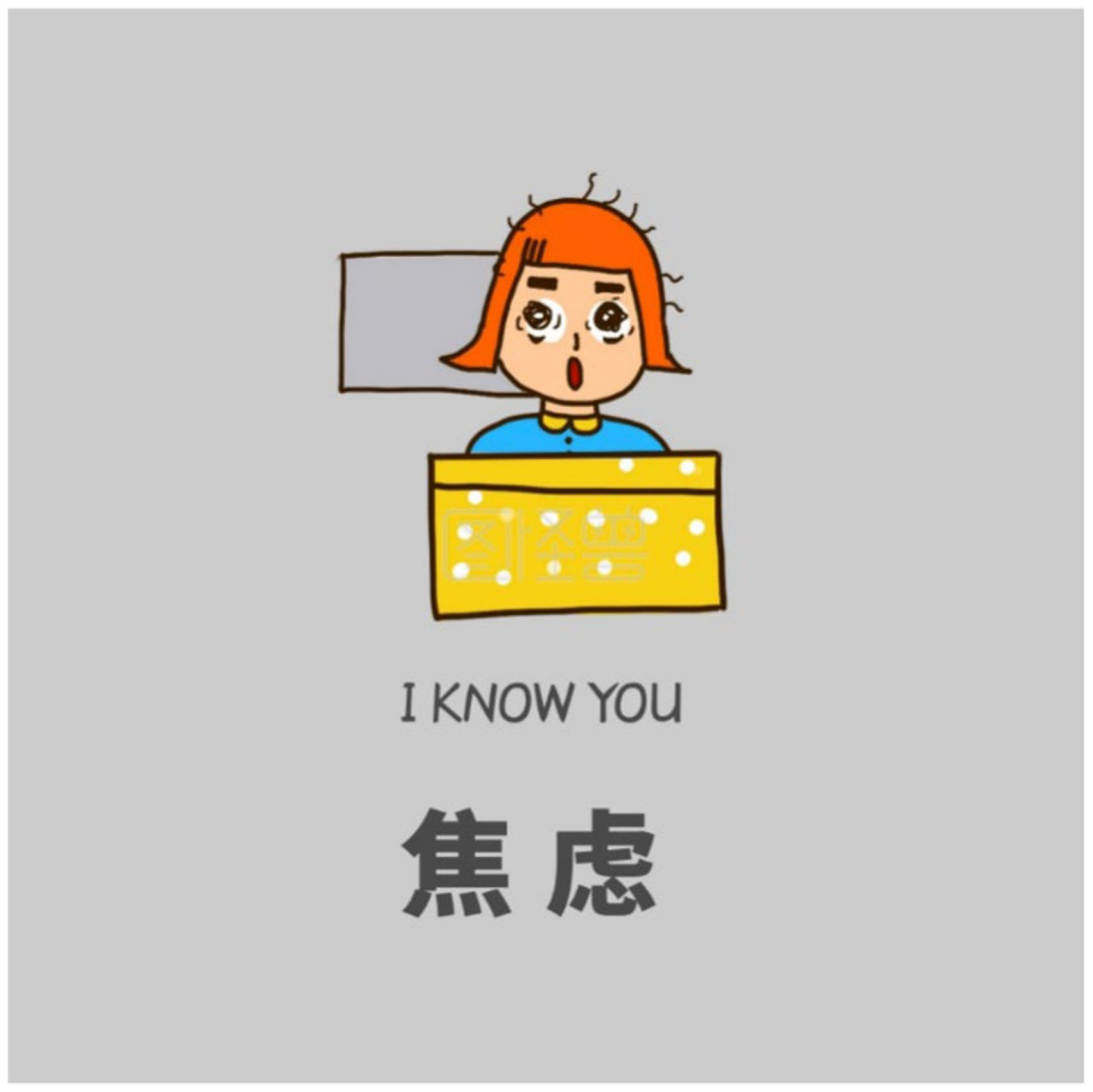
Anxiety disorders, academic stress, and future uncertainty.

**Figure 4:**
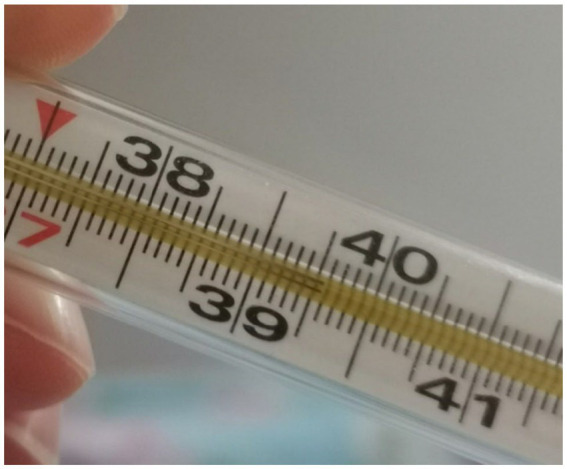
Illness, no income, and closure of residential area.

Based on the narratives of 170 participants, we identified eight major barriers to mental health during the COVID-19 restrictions, detailed in Table 4. The table categorizes the primary stressors, ranging from physical constraints to socio-economic disruptions. The accompanying visual results and narratives (e.g., Figures 3, 4) provide interpretive context for these barriers, visually representing the internal struggles of ‘academic pressure’ and the somatic reality of ‘infection and illness.’ Due to limited space, we present two representative examples that highlight the intersection of psychological anxiety and physical health.

**Table 4:** Barriers to mental health during COVID-19 (*N* = 170).

Barriers (8 main themes)	*N*	%
1. Restricted daily life	59	34.71
Lack of entertainment		
Disruption of life rhythms		
Powerlessness of life, out of control		
Limited sports venues		
Delivery not open for business		
Canceled flights		
Loss of freedom		
Emergency quarantine		
2. Economy	56	32.94
No income		
Depression		
Rising prices		
Unemployment and stoppages		
Massive bankruptcies of entity enterprises		
Delayed wage payments		
Payroll instability		
3. Epidemic prevention policy	41	27.12
Community closure		
Inappropriate scheduling of nucleic acid testing		
Repeated adjustment of resumption of work and production dates		
Epidemic quarantine at home		
Massive nucleic acid testing		
One-size-fits-all management		
Complicated leave of absence procedures		
City closure		
Repeated nucleic acid testing		
School closure		
4. Lack of social support	41	24.12
Non-cooperation of residents		
Rumor spreading		
Difficulty distinguishing between true and false information on the internet		
Lack of social activities		
Conflicts among family members		
Lack of psychological counseling		
Separation from family/lovers/friends		
Anti-social behavior of some patients		
Profiteers hoarding necessities		
Negative news		
Weakened sense of religious belonging		
5. Life and health issues	36	21.18
Fear of being infected with the virus		
Death of a family member		
Low resistance		
Threats to health		
Scarcity of medical resources		
Infection with COVID-19		
Vicarious traumatization		
Increased number of people infected by the epidemic		
Myopia		
Risk of infection		
6. Academic pressure	18	10.59
Examination		
Ineffective learning		
Unable to do regular classroom learning		
Ineffective learning in online classes		
Group reporting assignments		
Lack of study materials		
Difficulty of the lessons		
7. Housing conditions	15	8.82
Depressing housing environment		
Living alone		
Scarcity of necessities		
Not being able to breathe fresh air		
Depressing and dreary scenery		
Emergency meals are hard to eat		
Not being able to get close to nature		
Inconvenient access to information in old residential areas		
8. Time	13	7.65
Waste of time		
Time management		
Long queues		

212 respondents; 34 invalid; 8 repetitions; 170 valid responses. We combined the themes represented under 3% of the total number of responses (170) following the completion of analysis as suggested by OPV developers.

A participant’s submitted illustration depicting the overwhelming nature of the pandemic’s psychological toll. This visual result correlates with the barrier themes of Restricted Daily Life and Academic Pressure, capturing the internal confusion, career uncertainty, and isolation experienced by students confined to campus environments.

Participant 39 submitted the following story for the barrier:

Because of COVID-19, I was confined to campus with strict rules and restrictions on my daily activities, leading to heightened anxiety. This anxiety stemmed from academic pressures, career uncertainties, and future prospects. I felt confused and frustrated about my feelings of anxiety. These anxieties made me feel unable to predict my future and worried about whether I would be able to reach my desired goals and pursue my ideal career. For this reason, I tried to talk to my tutor, classmates, or counselor for advice and support to develop a more reasonable study and career plan. At the same time, I also sought to better cope with my anxiety by adjusting my time management and finding suitable learning methods to cope with stress. In conclusion, although the anxiety caused by COVID-19 has made me feel stressed and uneasy, I do believe that through a positive attitude and motivated actions, I can find solutions to my problems and gradually overcome my anxiety.

Participant 39 submitted the following summary text for the photograph and story:

Anxiety disorders, Academic stress, and Future uncertainty.

This photograph of a thermometer reading 39.5 °C serves as a stark visual indicator of the barrier theme Life and Health Issues. The image, coupled with the participant’s narrative, illustrates the compound trauma of physical COVID-19 infection exacerbated by strict home isolation, lack of community information, and economic anxiety.

Participant 147 submitted the following story for the barrier:

Access to information is extremely inconvenient as the residential area is quite old and families do not know about the community WeChat group, so they do not know when the neighborhood will be unblocked or when they will do the nucleic acid [test]. Secondly, it is difficult to obtain money because both mom and dad stay at home and have no source of money for a long time. Finally, there is the physical discomfort of COVID-19. The picture is of my temperature taken after I had a fever, which had reached 39.5 °C. After that, I measured it with the antigen self-test and found out that I was infected with the virus. It was very uncomfortable with a high fever, pain all over, headache, sore throat. The first 3 days were excruciating, and then I would slowly get better. All I could do in those 2 weeks was to stay at home, isolate myself well in my room, and disinfect everything I touched to keep from transmitting the virus to others.

Participant 147 submitted the following summary words for the photo and caption: Illness, No income, and Closure of residential area.

### 4.3 Attribution of facilitator and barrier themes to EST levels

After excluding incomplete responses and ambiguous answers, a total of 95 participants answered the attribution of facilitators and barriers at the EST level questions, summarized in Table 5.

**Table 5:** Attribution of facilitator and barrier themes to EST levels.

EST levels themes	Individual/Intrapsychic	Microsystem	Exosystem	Macrosystem	All together
Facilitator	70.53% (*n* = 67)	40.00% (*n* = 38)	46.32% (*n* = 44)	46.32% (*n* = 44)	36.84% (*n* = 35)
Barrier	64.21% (*n* = 61)	40.00% (*n* = 38)	47.37% (*n* = 45)	34.74% (*n* = 33)	41.05% (*n* = 39)

We allowed the participants to attribute the factors to more than one level; N = 95.

## 5 Discussion

Based on questionnaire data, we identified 12 main facilitators and 8 main barriers to participants’ mental health during the COVID-19 pandemic. In this section, we discuss the demographic information (Table 1), contextual factors (Table 2), main facilitators (Table 3), main barriers (Table 4), and ecological system theory levels (Table 5). Due to limited space, we kept our discussion to a limited number of findings across all results.

Before discussing specific demographic and contextual factors, it is essential to highlight how our proposed OPV approach improves upon existing baseline methodologies. Traditional baseline models in pandemic mental health research predominantly utilize standardized quantitative surveys (e.g., Likert scales) that quantify the prevalence of anxiety or depression but often strip away contextual depth. In contrast, our qualitative OPV approach performs significantly better in capturing the nuanced, multidimensional realities of the participants. For instance, while a baseline quantitative tool might merely register a high numerical score for “academic stress,” our visual data and SHOWED narratives (e.g., Figure 3) reveal the underlying mechanisms behind it—such as the intersection of future career uncertainty and strict spatial confinement. By doing so, this method empowers participants to highlight highly localized barriers and facilitators that predefined baseline surveys frequently miss, thereby providing a more granular and culturally sensitive evaluation.

### 5.1 Demographic information

In this survey, 87 participants (91.58%) had been diagnosed with COVID-19, which indicated that the impact of COVID-19 on the public during the outbreak was widespread. Regarding education level, most participants had a 4-year degree (76.84%, *n* = 73), followed by a master’s/doctorate degree (11.58%, *n* = 11). Regarding socioeconomic status, the largest number of participants had low income (50.53%, *n* = 48), which inextricably linked to the fact that the participants were students (47.37%, *n* = 45). Regarding religious belief, only three participants (3.16%) clearly stated or had religious beliefs when participating the study.

### 5.2 Discussion of contextual factors related to mental health during the pandemic

As shown in Table 2, the mean score of the participants’ level of internet access was 82.86 ± 20.22; the mean score of participants who had personal computers or tablets was 88.57 ± 25.35; and the mean score of participants who had smartphones was 95.31 ± 16.16. This suggests that the majority of the population had, to a large extent, tools to access the internet, but it is more important to consider whether the tools could be utilized to effectively access relevant resources (e.g., the mean score of participants using the internet to access mental health services or related information was 59.59 ± 32.61). Internet technology emanates from regions dominated by telecommunications infrastructure (56), which means the utilization rate of electronic products is also a symbol of the development of internet technology. Relevant studies have pointed out that the internet has disruptively changed the way that humans interact with the environment (57) and government’s responsiveness and behavior toward public needs (58). Therefore, the popularization of technology and electronic products is an important factor that needs to be paid attention in the development of modern mental health consulting services.

Similarly, our results show that the mean score of participants using offline mental health counseling services (mean: 29.84 ± 31.95) was lower than the mean score of participants using online mental health counseling services (mean: 32.11 ± 33.0). The emergence of information and communication technology has fundamentally changed the way people communicate with each other and utilize services to provide professional knowledge (e.g., psychological counseling) (59). With the support of this technological background, online counseling can place the time, location, speed, and direction of counseling under the control of customers (60). Therefore, the mean score of satisfaction with received or available online mental health counseling services was higher (43.58 ± 35.90). During the COVID-19 pandemic, the mean score of satisfaction with received or available offline mental health counseling services was lower (41.41 ± 35.09). The report results once again demonstrated that the epidemic inevitably led to a redefinition of our relationship approach, which was no longer based on proximity but rather on distance (the mean score of participants who practiced social distancing was 66.54 ± 30.69), and this definition is further emphasized due to the legitimacy of medical science (61).

In addition, the mean score of participants who identified themselves as spiritual people was 48.29 ± 34.25; the mean score of participants who identified themselves as religious people was 24.20 ± 26.89; and the mean score of participants who found religiosity/spirituality important in their life was 41.32 ± 33.45. This indicates a large gap with the data from Turkey (88, 91). However, Cheng et al. (62) have shown that religious beliefs have a therapeutic effect on “everyday function” and “emotions”; i.e., religious beliefs can indeed promote mental health.

### 5.3 Discussion of mental health facilitators during the pandemic

The study found about every three in 10 Chinese participants indicated that social support such as nucleic acid detection, medical resources, harmonious and warm atmosphere, volunteer service, mass cooperation, community and school support and so on were the main facilitators promoting mental health during the COVID-19 pandemic (Table 3). This finding is consistent with a study in Nigeria (85). Among these social supports, large-scale nucleic acid detection plays an important role in rapid diagnosis, treatment evaluation, and epidemic prevention and control as a unique tool in China’s epidemic prevention policy (63). Therefore, improving the ability of nucleic acid detection is important (64). Additionally, positive coping and good social support have a positive impact on people’s mental health (23). It has been emphasized that a person’s perception of social relationships and community support following a traumatic event has a protective effect on mental health (65).

Among participants, 15.26% reported that the second facilitator was socialization, such as interaction with friends, the company of family, participation in recreational activities, and studying with classmates (Table 3). Maintaining social distance is a new public mandate from COVID-19, but it also affects public mental health. Research has suggested that the COVID-19 pandemic changed people’s social-spatial relationships, which is associated with an increase of mental health problems, including fear, anxiety, etc. (66). Therefore, stable interpersonal relationships play an important role in maintaining and promoting mental health (67).

The third facilitating theme reported by participants was scenery (Table 3). A total number of 27 participants explicitly reported that good weather alleviated inner restlessness, the charm of nature made difficulties insignificant, and the happiness brought by enjoying this beauty contributed to mental health (68). This may be related to research on forest medicine – the impact of a forest environment (forest bathing) – on human health (69).

As the world grapples with the challenges brought by COVID-19, some participants reported that self-awareness and reflection (13.68%) and government support (11.58%) were the driving forces of promoting mental health (Table 3). In the questionnaire, participants expressed their pride in China, the Communist Party of China (CPC), and China’s epidemic prevention and control policies. This is the most distinct factor compared to other non-COVID-19 OPV mental health studies (90) and also COVID-19 OPV mental health studies (85, 90).

In addition, in our study, participants reported paying attention to entertainment (11.58%) and media (4.21%) and so on. These facilitators promoting mental health during the COVID-19 pandemic are similar to findings from other research (85).

### 5.4 Discussion of mental health barriers during the pandemic

In our findings, most reported themes were eight main barriers influencing mental health during the COVID-19 pandemic. Due to space limitations, we will only discuss a few major barriers.

Firstly, the review of the main themes determined in the study that complicated mental health during the COVID-19 pandemic revealed that the strongest theme was associated with movement restrictions or lockdowns (Table 4) which included a lack of entertainment, disruption of life rhythms, loss of freedom, inability to travel, and so on. (85) reported similar barriers in promoting and hindering mental health in Nigeria. Based on a review of the literature, Liu et al. (70) pointed out that restrictive measures such state blockades, maintaining social distance, and isolation, etc., have a greater negative impact on people’s psychology than a positive one.

Secondly, economic factors lead to some barriers to mental well-being in China that include a lack of income, unemployment and stoppages, and massive bankruptcies of entity enterprises (Table 4). Burns' (71) study showed that income inequality plays a mediating role in the relationship between poverty and mental illness which indicated that poor economic conditions are important factors leading to psychological problems.

Thirdly, the Chinese government’s prevention and control policies during the pandemic had alleviated the spread of the virus and provided effective protection. However, school and community closures, and one-size-fits-all management, etc. had led to inconvenience in movement and complex leave procedures. OPV studies in other countries had similar results (86, 87, 89).

Finally, the study participants reported vicarious traumatization, viral infection, death of patients, and physical health, etc. during the COVID-19 pandemic as barriers to their mental health (Table 4). Research has shown that COVID-19 patients, healthcare professionals, and the general population had poor mental health conditions such as post-traumatic stress, depression, and anxiety (72). This may lead to a shortage of healthcare personnel or services. Therefore, it is possible to consider telepsychiatry and online services to evaluate patients (73).

### 5.5 Discussion of attribution of facilitator and barrier themes to EST levels

The participants attributed their facilitating factors for mental health during the pandemic to the individual/intrapsychic 70.53% (*n* = 67), exosystem 46.32% (*n* = 44), and macrosystem 46.32% (*n* = 44). In other words, participants believed their source of mental health strength was linked to their efforts, social power (e.g., media, social service, etc.) and macro-environment (e.g., government and policies, economy, etc.). These findings supported research asserting that financial problems can explain about 5% of negative emotions in the context of stable economy and universal health care (74). During the unprecedented COVID-19 pandemic, the ability to respond to global changes in daily life in a sustainable way depends on oneself, who as an individual with mental health and physical health can consciously and actively focus on psychological happiness (75).

While barriers were mostly attributed to the individual/intrapsychic level 64.21% (*n* = 61), this differs from other research findings. Psychological health research has emphasized that participants viewed all factors of EST as challenges (85). Distance education research also indicated that students view the barriers to online distance education from multiple perspectives and attributed them to all system categories (87). However, our research participants believed that individual/intrapsychic factors (such as emotions, thoughts, behaviors, etc.) were the most important barriers affecting mental health.

### 5.6 Limitations

Several methodological and sampling limitations contextualize the findings of this study. First, regarding the Online Photovoice (OPV) approach, the reliance on participant-generated visual and narrative data (via the SHOWED model) introduces inherent self-selection bias; participants actively curate the realities they choose to document. Furthermore, the qualitative nature of Online Interpretative Phenomenological Analysis (OIPA) relies on the researchers’ putting the themes based on the participants submitted key summary words and their stories, which includes a piece of hermeneutic interpretation. This means that despite rigorous OIPA steps, thematic extraction cannot be entirely divested from subjective lenses.

Second, the deployment of an online survey structurally excludes populations facing digital marginalization, particularly older adults or individuals lacking smartphone access. Finally, the sample is predominantly drawn from higher education cohorts within Sichuan Province. Rather than invalidating the findings, this geographic and demographic concentration provides valuable, localized insights into the lived experiences of young adults in China’s inland and developing regions. However, it necessitates academic caution when extrapolating these phenomenological insights to other demographic groups or economically disparate coastal provinces.

### 5.7 Implications

Our findings provide important information for intervening in the mental health of individuals in response to the negative impact of the COVID-19 pandemic. We offer the following implications for researchers, mental health providers, key authorities and advocates, and educators.

#### 5.7.1 For researchers

Researchers could use OPV to further elucidate the results that emerged from this study (e.g., social support, China’s epidemic prevention and control policies, etc.) to develop a deeper understanding of mental health interventions for Chinese participants. For example, previous studies have suggested that healthcare workers in China may have had poorer mental health during COVID-19 compared to the general population (21, 23), and through an OPV approach, this population may be able to provide us with their unique facilitators and barriers. Secondly, researchers may consider using the OPV method in conjunction with other empirical research methods to provide a more comprehensive perspective on the research question. For example, used in conjunction with methods such as traditional regression, qualitative data obtained in OPV methods can be quantitatively analyzed to provide deeper insights. Finally, building positive partnerships is critical to the successful dissemination of OPV methods (52, 90). Building good partnerships with communities, educational institutions, and stakeholders can help researchers integrate better into the community and gain the trust and support of participants (76).

#### 5.7.2 For mental health providers

Our study explored facilitators and barriers to mental health during COVID-19 among Chinese participants, revealing the critical role of social support and friends in mental health promotion, while also highlighting the adverse effects of daily life limitations and financial stress on mental health. These findings provide useful insights for mental health providers. On the one hand, mental health providers should recognize the key role of social support in mental health promotion. Social support includes not only support from family and friends but also support from the community, healthcare organizations, volunteers, and the government (77, 85, 89). In the face of the stress and uncertainty of public health emergencies, building a solid social support network can help individuals cope better with challenges, reduce emotional stress, and enhance psychological resilience (78, 95). On the other hand, mental health providers need to understand the impact of daily life limitations and financial issues on mental health. As a result of the epidemic, individuals may face interruptions to their recreation and daily routines, as well as financial stress (12). Mental health providers should assist individuals with coping strategies and counseling to help them adapt to their new lifestyle and find alternative forms of recreation and relaxation to reduce psychological stress and anxiety.

#### 5.7.3 For key authorities and advocacy

Based on our findings, we make the following recommendations to the government and authorities to better safeguard the public’s mental health well-being during public health emergencies. First, the government should strengthen the social support system. Given the important role of social support in mental health promotion, the government can provide timely psychological support and counseling to individuals by setting up mental health hotlines and establishing community mental health centers (79, 90). Second, the government can introduce economic support policies to reduce economic stress. One of the important findings of this study is that economic problems such as unemployment and decreased income of individuals due to COVID-19 can harm their mental health. The government can take measures such as providing unemployment benefits and granting temporary subsidies to help individuals through difficult economic times and reduce their psychological stress (80). Finally, the government can conduct mental health advocacy and education initiatives to raise the public’s awareness of mental health. By organizing online lectures on mental health and releasing information on mental health, the government can help the public understand mental health issues better, eliminate stigmatization of mental health problems, and encourage individuals to proactively seek mental health support (81, 82, 87), which will promote the overall level of mental health in society.

## 6 Conclusion with future work

In summary of our key findings, framed through the Ecological Systems Theory (EST) and Community-Based Participatory Research (CBPR) paradigms (51), our findings indicate that pandemic-era mental health is largely dictated by the friction between individual intrapsychic resilience and macrosystemic constraints. While social support networks and targeted government interventions acted as primary psychological facilitators, strict mobility restrictions and the associated economic precarity emerged as the dominant systemic barriers.

Regarding the main contributions, this study diverges from conventional, psychometric evaluations by employing an Online Photovoice (OPV) methodology to capture the lived experiences of Chinese participants during the COVID-19 pandemic. By shifting the paradigm from participants as passive subjects to active co-investigators, we elicited highly localized coping mechanisms—such as community-based volunteering and micro-adaptations to lockdown protocols—that top-down epidemiological tools frequently overlook. This approach aligns with foundational participatory research principles that prioritize community.

To advance this line of inquiry, future research must address current methodological constraints. Future studies should adopt mixed-method frameworks to bridge the digital divide, potentially integrating offline participatory action research to include digitally marginalized populations (76). Crucially, given the established bidirectional relationship between psychological distress and biological disruption—such as pandemic-induced sleep architecture degradation and insomnia (48, 49) subsequent studies should triangulate OPV’s subjective visual narratives with objective physiological data. Combining the subjective qualitative narratives derived from OPV with objective, real-time physiological data will enable a precise, multidimensional understanding of pandemic-induced trauma. For instance, recent advancements in sensor-based technologies and Internet of Things (IoT) devices have demonstrated significant efficacy in problem detection, real-time health monitoring, and individual protection during the COVID-19 pandemic (83, 84). Integrating these advanced measurement tools alongside machine learning classifiers (50) will provide robust, triangulated data for early-warning systems and targeted, technology-assisted interventions in future public health crises.

## Data Availability

The original contributions presented in the study are included in the article/supplementary material, further inquiries can be directed to the corresponding author.
